# Skin temperature influence on transcutaneous carbon dioxide (CO_2_) conductivity and skin blood flow in healthy human subjects at the arm and wrist

**DOI:** 10.3389/fphys.2023.1293752

**Published:** 2024-01-23

**Authors:** Emmanuel Dervieux, François Guerrero, Wilfried Uhring, Marie-Agnès Giroux-Metgès, Michaël Théron

**Affiliations:** ^1^ Biosency, Cesson-Sévigné, France; ^2^ EA4324-ORPHY, Univ Brest, Brest, France; ^3^ ICube, University of Strasbourg and CNRS, Strasbourg, France; ^4^ Explorations Fonctionnelles Respiratoires, Centre Hospitalier Régional et Universitaire de Brest, Brest, France

**Keywords:** carbon dioxide, ptCO_2_, tcpCO_2_, transcutaneous, exhalation rate, diffusion, blood flow, hyperaemia

## Abstract

**Objective:** present transcutaneous carbon dioxide (CO_2_)—tcpCO_2_—monitors suffer from limitations which hamper their widespread use, and call for a new tcpCO_2_ measurement technique. However, the progress in this area is hindered by the lack of knowledge in transcutaneous CO_2_ diffusion. To address this knowledge gap, this study focuses on investigating the influence of skin temperature on two key skin properties: CO_2_ permeability and skin blood flow.

**Methods:** a monocentric prospective exploratory study including 40 healthy adults was undertaken. Each subject experienced a 90 min visit split into five 18 min sessions at different skin temperatures—Non-Heated (NH), 35, 38, 41, and 44°C. At each temperature, custom sensors measured transcutaneous CO_2_ conductivity and exhalation rate at the arm and wrist, while Laser Doppler Flowmetry (LDF) assessed skin blood flow at the arm.

**Results:** the three studied metrics sharply increased with rising skin temperature. Mean values increased from the NH situation up to 44°C from 4.03 up to 8.88 and from 2.94 up to 8.11 m·s^−1^ for skin conductivity, and from 80.4 up to 177.5 and from 58.7 up to 162.3 cm^3^·m^−2^·h^−1^ for exhalation rate at the arm and wrist, respectively. Likewise, skin blood flow increased elevenfold for the same temperature increase. Of note, all metrics already augmented significantly in the 35–38°C skin temperature range, which may be reached without active heating—*i.e.* only using a warm clothing.

**Conclusion:** these results are extremely encouraging for the development of next-generation tcpCO_2_ sensors. Indeed, the moderate increase (× 2) in skin conductivity from NH to 44°C tends to indicate that heating the skin is not critical from a response time point of view, *i.e.* little to no skin heating would only result in a doubled sensor response time in the worst case, compared to a maximal heating at 44°C. Crucially, a skin temperature within the 35–38°C range already sharply increases the skin blood flow, suggesting that tcpCO_2_ correlates well with the arterial paCO_2_ even at such low skin temperatures. These two conclusions further strengthen the viability of non-heated tcpCO_2_ sensors, thereby paving the way for the development of wearable transcutaneous capnometers.

## 1 Introduction

Due to its clinical significance, continuous monitoring of the arterial CO_2_ partial pressure—paCO_2_—is of paramount importance in medical practice, especially for patients presenting severe respiratory disorders (Wagner, 2015). The gold standard to get a single paCO_2_ reading consists in an arterial puncture followed by a gaseous analysis of the collected blood sample. Unfortunately, this procedure—tersely referred to as the “blood gases” in clinical settings—is both painful and risky (Scheer et al., 2002), requiring trained personnel as well as expensive blood gas analysers. It also calls for a quick analysis of the blood samples following their collection, which adds stress to hospital logistics (Nanji and Whitlow, 1984). These major drawbacks led to the development of transcutaneous CO_2_ monitors, which consist in a Stow-Severinghaus electrode—mainly a pH-meter bathing in a bicarbonate solution—heated in the 41–44°C range, and placed against a patient’s skin (Severinghaus and Astrup, 1986; Huttmann et al., 2014). This electrode measures a transcutaneous CO_2_ partial pressure—the tcpCO_2_—which correlates well with the paCO_2_ if the skin is heated above at least 38°C (Wimberley et al., 1985).

Yet, these monitors also suffer from several weaknesses: *i)* their important drift requires a recalibration with an appropriate gas mixture every 8 hours, at most (Bendjelid et al., 2005), *ii)* the thin membrane covering the electrode is fragile, and needs to be replaced every 2 weeks or so (Lermuzeaux et al., 2016), *iii)* the heating power required by the electrode to maintain the skin in the above-mentioned temperature range is about 100–200 mW^1^, thus precluding its use in a battery-powered wearable stand-alone device and *iv)* their elevated price tag prevents their widespread use, would it be in a clinical or home-based setting. Consequently, the development of an alternative to the existing tcpCO_2_ monitors appears mandatory, and has been an active research field in the last decades (Dervieux et al., 2022; Section 4.1).

Recently, in a review article aiming at encompassing the diversity of CO_2_ measurement techniques with a focus on biomedical applications, we divided the issue of developing such an alternative tcpCO_2_ monitor into three research areas (Dervieux et al., 2022):

1. Due to the above-mentioned drift, and high cost of the Stow-Severinghaus electrode, an alternative CO_2_ *measurement technique* is needed.
2. Then, in order to dimension the sensor-to-be, it is essential to accurately know the CO_2_ *exhalation rate* through the skin, as the latter directly influences the response time of the sensor.
3. Finally, it is mandatory that the tcpCO_2_ and paCO_2_ are in good agreement at the skin temperature considered for measurement. *Id est*, that the *correlation* between tcpCO_2_ and paCO_2_ is satisfactory at the latter temperature.

Regarding the first point, it appeared to us that, among the many technologies reviewed, a polymer patch embedding a CO_2_-sensitive fluorophore would be particularly advisable. Interestingly, this trail has recently been followed by Cascales et al. (2022) or Tufan and Guler (2022) with some success, although no *in vivo* experiment have been conducted to date. This point will be the object of future studies and is not developed any further in this paper.

The second and third points, on the contrary, are at the very heart of the present study. Starting with the exhalation rate, the main issue with data available in the literature—see Table 3—is that the skin temperature is only mentioned once—by Eöry (1984)—and never accurately regulated when this parameter is measured (Fitzgerald, 1957). Even though Levshankov et al. (1983) crafted a heating device, they do not report the temperature setpoint that they used. Thus, the present study aims at filling this gap by measuring the influence of skin temperature on the transcutaneous CO_2_ exhalation rate.

While the tcpCO_2_/paCO_2_ correlation is excellent at—or above—42°C (Conway et al., 2018), scarce are the authors who investigated lower skin temperatures, with none but Wimberley et al. (1985) experimenting with temperatures as low as 38°C. The reason for heating the skin in the first place is to trigger a local reactive hyperaemia (Roustit and Cracowski, 2012). By doing so, the subcutaneous tissues are flushed with fresh arterial blood, and their gaseous content thus gets closer to the arterial one (Koch, 1965; Rooth et al., 1987; Zavorsky et al., 2007). While temperatures in the 42–44°C range have often been used to trigger maximal hyperaemia, lower temperatures have been seldom explored (Hodges et al., 2016), and we thus took advantage of our exhalation rate measurements to measure the skin blood flow at lower temperatures simultaneously.

Then, the reader should bear in mind the importance of skin temperature for designing a new kind of tcpCO_2_ sensor. Indeed—ideally—such a sensor should heat the skin as little as possible for two main reasons: *i)* heating the skin is uncomfortable for the patient and can wake them up in the case of night time monitoring, and *ii)* it consumes a significant amount of power, which precludes using such sensor in a wearable, as mentioned above. Yet, such an unnoticeable and wearable tcpCO_2_ sensor would be highly desirable in a telemonitoring context for home use, reducing the need for hospital visits. Indeed, if the positive impact of tcpCO_2_ telemonitoring is yet to be demonstrated—for the obvious reason that the corresponding wearable tcpCO_2_ monitor does not exist at the time being—several clinical trials demonstrate the beneficial contribution of telemedicine—*a.k.a.* telehealth—on both patient’s outcome and costs of admission in a variety of conditions (Steventon et al., 2012; Yun et al., 2018; Kruse et al., 2019). Additionally, the outbreak of contagious pandemics—such as COVID-19 (Garfan et al., 2021)—and the rapid development of the health wearable market (Dunn et al., 2018; Yetisen et al., 2018; Chung et al., 2019; Dagher et al., 2020) may also promote the use of telemonitoring in medical practice. For these reasons, *not* heating the skin while measuring tcpCO_2_ would be highly desirable.

The following study focuses on measuring the transcutaneous CO_2_ exhalation rate and cutaneous micro-circulation on the full NH–44°C skin temperature range. Two measurement sites were investigated: the dorsal side of the wrist and the lateral aspect of the upper arm, while the skin blood flow was only measured at the upper arm. Additionally, a strong emphasis was placed on the transcutaneous CO_2_ *conductivity*, which may be preferred to the well-known exhalation rate because of its more intrinsic nature—in particular, the latter conductivity does not depend on the ambient CO_2_ level, nor on the subject’s paCO_2_, as opposed to the exhalation rate, which is influenced by both.

## 2 Materials

### 2.1 The transcutaneous CO_2_ rate sensor

**FIGURE 1:**
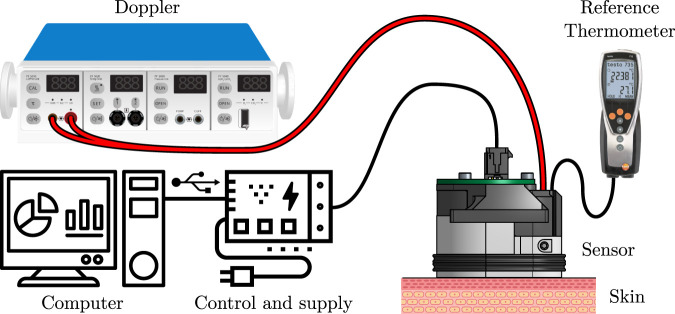
General outline of the rate sensor and its peripherals. See the text for further explanations.

A custom transcutaneous CO_2_ diffusion rate sensor—hereafter simply denoted as “the sensor”—was developed for the needs of this study. Its basic working principle is close to that evoked by Dervieux et al. (2022)—and will be further detailed in Section 3.1.1—while its design is inspired by the early works of Eletr et al. (1978) and Greenspan et al. (1981). The general outline of the sensor and its peripherals can be seen in Figure 1.

**FIGURE 2:**
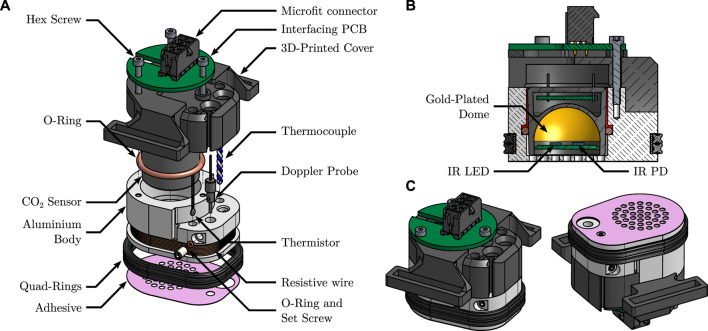
Detailed views of the sensor. **(A)**: exploded view, detailing its different parts. **(B)**: cut view, showing the inner functioning principle of the CO_2_ sensor. Note the epoxy resin sealing, in red. **(C)**: isometric views from above, and below, of the fully assembled sensor, illustrating the grid-shaped sensor’s sole.

The sensor, designed to be placed against the subject’s skin by mean of a double-sided adhesive, is connected to three main apparatuses: *i)* a calibrated, reference thermometer (Testo 735, Testo, Germany) equipped with a type K thermocouple (110-4482, RS Pro, United Kingdom), *ii)* a Doppler perfusion monitor (Periflux 5000, Perimed, Sweden) equipped with a 407 probe, and *iii)* a control and supply block, consisting in a thermostat, a power supply unit, and a Universal Serial Bus (USB) to universal Asynchronous Receiver Transmitter (UART) converter, embedded in a 3D-printed case. For the sake of conciseness though, the control and supply block is only detailed in the Supplementary Material S1, which also contains a thorough analysis of the safety issues that may arise when using this sensor. The sensor itself can be seen in great details in Figure 2. It consists in an aluminium (2017A) body, which serves as a support for the following elements: a CO_2_ sensor, a heating resistive wire, a thermistor, a thermocouple, the Doppler probe, a poly-lactic acid (PLA) 3D-printed cover, and an interfacing Printed Circuit Board (PCB). Complete drawings of the sensor’s body are provided in the Supplementary Material S1.

The CO_2_ sensor is a MinIR (ExplorIR–M5%, CO_2_Meter, United States), an off-the-shelf, compact, Non Dispersive Infra-Red (NDIR) CO_2_ sensor, with a full range of 5% and an accuracy of 70 ppm ± 5% of reading at Standard Temperature and Pressure (STP)—see Hodgkinson and Tatam (2012) for further details on the operating principle of such sensors. Its internals—a pair of IR Light Emitting Diode (LED) and photodiode (PD) facing a spherical, gold-plated mirror—may be seen in the cut view of Figure 2. The pCO_2_ inside the sensor was recorded with a sampling frequency of 2 Hz.

As the gas-tightness of the measuring chamber is a critical aspect of the sensor’s operating principle—see Section 5.1.3—a two-stage sealing was implemented: *i)* a silicone-grease coated (515520, GEB, France), soft—60 Shore A hardness—silicone O-ring was placed between the aluminium body and the CO_2_ sensor itself and *ii)* liquid epoxy resin (Résine Cristal, Gédéo, France) was cast in the remaining interstice between the latter two elements—illustrated in the cut view of Figure 2 in vivid red.

The thermoregulation of the sensor is performed by mean of a resistive wire for heating, coupled to a thermistor for temperature measurement and regulation. For verification purpose, an additional thermocouple was also added, as stated above. The heating wire consists in 2 × 15 turns of 28 Ω·m^−1^, 0.15 mm in diameter, enamelled, resistive, constantan wire (Isotan, Thomsen), connected in parallel. The wire delivers a total heating power of 6.1 W under 12 V, and is coiled around the aluminium body, in a dedicated groove. The bottom of the groove is covered with a layer of 0.25 mm thermally conductive double sided tape (8810, 3M, United States) prior to coiling the wire, and the latter is finally covered with two nitrile quad rings, as can be seen in Figure 2. This covered layout prevents burns caused by direct contact with the heating wires. The thermistor (151-237, RS Pro, United Kingdom) and thermocouple were glued in two dedicated flat-bottom mounting holes which were pre-filled with a thermally conductive, electrically non-conductive, epoxy resin (8329TFM, MG Chemicals, Canada). Care was taken that *i)* the distance between the bottom of the mounting holes and the heating wire and *ii)* that between the sole of the sensor’s body and the heating wire were equal, in order to ensure that the temperature measured by the thermistor and thermocouple is close to that of the skin.

The Doppler probe is housed in a dedicated hole, and can slide vertically, in such a way that it can be adjusted to outcrop the sole of the sensor, coming in direct contact with the skin. It holds in place by mean of a cup-pointed, headless, set screw which compresses it radially via an O-ring, so as not to damage the probe. The raw Doppler perfusion signal—originally sampled at 62.5 Hz—was downsampled to 0.625 Hz and low-pass filtered using a tenth-order Butterworth filter prior to further analysis—see Section 3.1.2.

**FIGURE 3:**
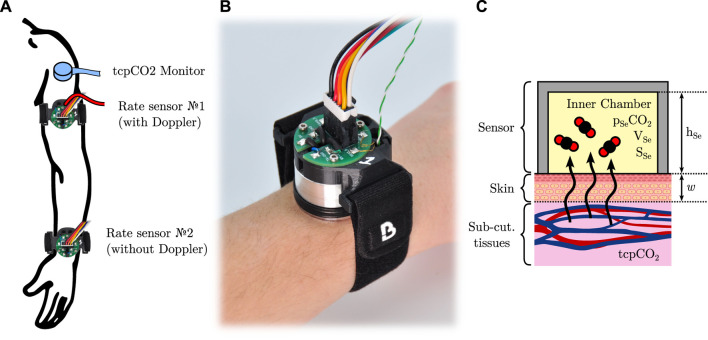
**(A)**: outline of the sensors used in this study, and their location. **(B)**: a picture of the sensor with its connection cable assembly and strap, attached on a wrist. **(C)**: simplified model of CO_2_ diffusion through the skin inside a closed sensor.

The 3D-printed cover and interfacing PCB were added for usability purposes: the 3D-printed PLA cover allows to attach a strap (HTH 833 with H83 hooks, Velcro, United Kingdom) to the sensor in order to maintain it against a subject’s skin, as illustrated in Figure 3B, while the interfacing PCB gathers the four UART pins from the CO_2_ sensor, the two ends of the thermistor, and those of the heating wire into a single eight-pins Microfit connector (0430450812, Molex, United States).

The adhesive itself consists in a disposable laser-cut, double-sided, clinical-grade tape (1567, 3M, United States). For ease of application, a special tooling was developed to accurately align the sensor and the adhesive together—see Supplementary Material S1.

### 2.2 Reference tcpCO_2_ monitor

In addition to the above-detailed custom-made sensor, a clinical-grade tcpCO_2_ monitor (TCM4, Radiometer, Denmark) was also used on the upper deltoid—one of the recommended sites for tcpCO_2_ monitoring (SenTec, 2016)—yielding a continuous reference tcpCO_2_ reading. The tcpCO_2_ sensor itself (tc Sensor 54) was affixed to the skin using an appropriate attachment ring and contact gel, and it was re-membraned and re-calibrated when needed, as per the manufacturer’s guidelines. All the accessories used to this end were Radiometer’s (Radiometer, 2020).

### 2.3 Sensors positioning

The different sensors and measurement sites chosen in the study are illustrated in Figure 3A. All sensors were placed on the subject’s left arm: the reference tcpCO_2_ monitor was placed on the upper deltoid, as mentioned above, while two custom rate sensors were positioned as follows. The first one was equipped with the Doppler probe, and was attached on the distal side of the upper arm, immediately below the deltoid, at the junction point between the upper part of the biceps, the lower end of the deltoid, and the triceps. The second rate sensor was placed on the dorsal side of the wrist, and did not include a Doppler probe. Both sensors were affixed to the subject’s skin by mean of the above-mentioned double-sided adhesive, and secured in place with a Velcro strap. Additionally, the subject’s arm laid comfortably onto an arm gutter so that it remains still and relaxed for the whole duration of the experiments.

## 3 Methods

### 3.1 Measured metrics

#### 3.1.1 Skin CO_2_ conductivity and exhalation rate

The rate of diffusion—*a.k.a.* the exhalation rate—of CO_2_ through the skin per unit of area—hereafter noted *Q*, of dimension L^3^·L^−2^·T^−1^—can be measured by affixing to the skin a cup-like device, which entraps the skin-exhaled CO_2_. In this situation, the CO_2_ diffusion through the skin can be modelled as presented in Figure 3C. Briefly, the skin is considered as a CO_2_-permeable membrane of thickness *w* and diffusivity *D* towards CO_2_ (unit of m^2^ s^−1^). The partial CO_2_ pressure inside the sub-cutaneous tissues and inner chamber are tcpCO_2_ and *p*_*Se*_ *CO*_2_, respectively, and the sensor area in contact with the skin is *S*_*Se*_, while its equivalent height and volume are *h*_*Se*_ and *V*_*Se*_. It can be shown under certain hypotheses (Dervieux et al., 2022) that:

$$\frac{dp_{Se}CO_{2}}{dt}=\frac{D}{w\cdot h_{Se}}\cdot \left(tcpCO_{2}-p_{Se}CO_{2}\right)$$ (1)

Leading to a first-order behaviour for *p*_*Se*_ *CO*_2_, given by:

$$\begin{array}{ll} p_{Se}CO_{2}\left(t\right) & =tcpCO_{2}\cdot \left(1-e^{-\frac{t}{\tau }}\right)\\ & \;+p_{Se}CO_{2}\left(t=0\right)\cdot e^{-\frac{t}{\tau }},\;\text{with }\tau =\frac{h_{Se}\cdot w}{D} \end{array}$$ (2)

And *Q* is then equal to:

$$Q\left(t\right)=\frac{D}{w\cdot P_{0}}\cdot \left(tcpCO_{2}-p_{Se}CO_{2}\left(t=0\right)\right)\cdot e^{-\frac{t}{\tau }}$$ (3)

wherein *P*_0_ is the total atmospheric pressure at measurement site. However, since *Q* depends on both the ambient level of CO_2_—*via* *p*_*Se*_ *CO*_2_(*t* = 0)—and the subject’s capnia—*via* *tcpCO*_2_—we introduced the skin *conductivity*—from the thermodynamic or electrical analogy—expressed in m·s^−1^, and defined as:

K=Dw=P0⋅Qt=0tcpCO2−pSeCO2t=0 (4)

Contrary to *Q*, *K* is an intrinsic property of the skin and is independent of the tcpCO_2_/*p*_*Se*_ *CO*_2_ gradient. Additionally, deriving Eq. 2 and evaluating it at *t* = 0 yield:

$$K=\frac{h_{Se}}{tcpCO_{2}-p_{Se}CO_{2}\left(t=0\right)}\cdot {\left.\frac{dp_{Se}CO_{2}}{dt}\right|}_{t=0}$$ (5)

In practice, *K* was thus measured as follows, choosing arbitrarily *t* = 0 at each temperature change:

– *h*_*Se*_ is known by dividing *V*_*Se*_ by *S*_*Se*_. The latter is known by construction of the sensor’s aluminium body, while *V*_*Se*_ was estimated by filling a clone of the sensor with a low viscosity fluid—pure ethanol—and weighting it.
– the subject’s tcpCO_2_ was measured using the above-mentioned reference medical grade monitor. The extraction of a single tcpCO_2_ reading for each temperature set-point is detailed in Supplementary Material S1.
– *p*_*Se*_ *CO*_2_(*t* = 0) could be measured with a simple reading of the CO_2_ sensor.
– ${\left.\frac{dp_{Se}CO_{2}}{dt}\right|}_{t=0}$ was estimated by fitting a linear regression on the measured *p*_*Se*_ *CO*_2_. The latter regression was performed on the *p*_*Se*_ *CO*_2_ data starting 3 min after a temperature change—to allow for temperature homogenisation of the different inner parts of the sensor—and up to 18 min after, for a total of 15 min of data. This duration was chosen following a preliminary study performed on ten subjects, which yielded *R* ^2^ regression scores above 0.95 for a regression duration above 700 s (about 12 min).

In summary, the diffusion of CO_2_ through the skin was quantified by the skin CO_2_ conductivity *K*, which was measured at five different temperatures, each temperature corresponding to a 18 min measurement window for a total of 90 min of acquisition per subject, as detailed in Section 3.2, below. Additionally, Equation 4 was used to compute the corresponding equivalent *Q* (*t* = 0) with *p*_*Se*_ *CO*_2_(*t* = 0) ≈ 0—*i.e.* the skin CO_2_ exhalation rate in free air as commonly referred to in the literature. Of note, this *Q* (*t* = 0) was **not** observed in practice, since the sensor was left in place—and thus *p*_*Se*_ *CO*_2_(*t* = 0) ≠ 0 for most temperatures. For the sake of conciseness, in the remainder of this article, the letter *Q* alone or the mention of “skin CO_2_ exhalation rate” without further indications always designate the above-mentioned *Q* (*t* = 0). Finally, it should be noted that these *Q* values were derived mainly as a mean to compare with the existing literature, and that the actual statistical analyses were performed on *K*—see Section 3.3.

#### 3.1.2 Skin blood flow

**FIGURE 4:**
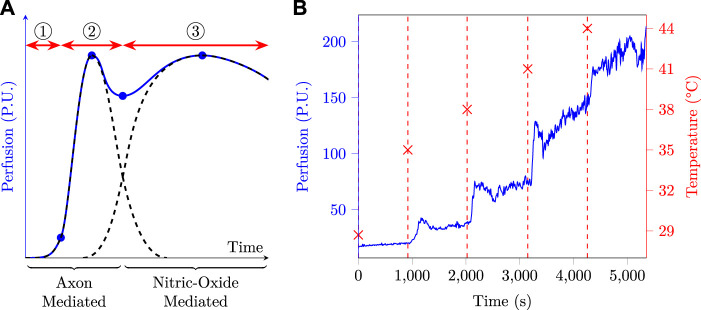
**(A)**: typical skin perfusion response to local heating, based on data from different sources (Minson et al., 2001; Cracowski et al., 2006; Minson, 2010; Frantz et al., 2012; Roustit and Cracowski, 2012). If we suppose the heat stress to be applied at time origin, three phases are usually observed: ① an onset lag corresponding to *i)* the heating time required by the sensor to reach its set-point temperature and *ii)* the time taken by the inner skin layers to reach this temperature and trigger the axon-mediated hyperaemia. ② the axon-mediated hyperaemia which rises quickly and then fades away. ③ the nitric-oxide mediated hyperaemia, whose onset is slower and which slowly fades away if the temperature set-point is not too elevated. **(B)**: perfusion and skin temperature as a function of time as measured at the arm of a test subject.

The skin blood flow—*a.k.a.* (sub)cutaneous micro-circulation or perfusion—was measured using LDF, and expressed in Perfusion Units (P.U.), a dimensionless arbitrary unit that reflects both the amount and the speed of moving elements—mainly erythrocytes—seen by the Doppler probe (Bonner and Nossal, 1990). When the skin temperature rises, perfusion increases, a phenomenon known as heat-triggered—or thermal—reactive hyperaemia (Minson, 2010), whose dynamics is illustrated in Figure 4A. The respective durations of phases ①–③ were not specified in abscissa since they may vary markedly depending on the heating rate and temperature (Magerl and Treede, 1996; Del Pozzi et al., 2016). To give the reader an order of magnitude, phase ① usually lasts a few minutes, phase ② from 5 up to 10 min, and phase ③ from 30 up to 60 min (Minson et al., 2001; Cracowski et al., 2006; Minson, 2010; Frantz et al., 2012; Roustit and Cracowski, 2012).

Such a behaviour calls for some kind of data processing to yield a single representative perfusion metric for the initial bump, after-bump nadir, and final plateau. In this paper, SkBF_90_(T) was defined as the 90th percentile of the measured skin blood flow—SkBF—on an 18 min window at temperature T. This choice was made following preliminary measurements at the arm, an example of which is plotted in Figure 4B. The latter clearly exhibits five perfusion plateaux corresponding to the five temperature set points, and one can also distinguish a small initial bump at the onset of a new temperature, which is especially visible at 35, 38 and 41°C. Due to *i)* the high variability exhibited by the measured perfusion, especially at high temperature, *ii)* the fact that the nitric-oxide phase can take up to 30–40 min to establish (Barcroft and Edholm, 1943; Taylor et al., 1984; Minson et al., 2001; Frantz et al., 2012; Del Pozzi et al., 2016), and *iii)* the fact that each temperature window lasts 18 min, it seemed a good strategy to choose a metric which was more robust than the mean—*e.g.* the *n*th percentile—and focused on the very end of the observation window. In this regard, SkBF_90_(T) seemed to meet the latter requirements and was therefore chosen as perfusion metric. It was additionally normed by the maximal perfusion measured on a given subject—*i.e.* the SkBF_90_ value measured at 44°C. Finally, the LDF metric used throughout this study is thus:

nSkBF90(T)=SkBF90(T)SkBF90(44°C) (6)

### 3.2 Protocol design

The clinical study was interventional, monocentric, and involved 40 healthy human subjects. Inclusion criteria were an age between 18 and 80, and having given a free and informed consent. Exclusion criteria were the presence of cutaneous lesions at the measurement sites or skin conditions such as dermatitis or psoriasis, and taking vasodilator therapy. The research was approved by the local ethics committee (Comité de protection des personnes Sud-Méditerrannée II, IDRCB ref.: 2020-A02185-38), registered on Clinical Trials (NCT05637138), and it was carried out in accordance with the declaration of Helsinki.

After a preliminary visit during which the subjects were informed of the study modalities and gave consent, all measurements were performed during a single visit, during which the subjects were seated. This visit began with a visual inspection of the measurement sites for detection of cutaneous lesions. The sites were then shaved if needed for a good adhesion of the sensors, using an electric trimmer in order to avoid skin inflammation. The skin was then degreased and cleaned using isopropyl alcohol, and the three above-mentioned sensors were attached to their respective measurement sites. These preliminary steps also allowed for subject acclimation and lasted about 5 min. The measurement itself then began, consisting of five 18 min periods, corresponding to five temperatures for the two rate sensors: NH, 35, 38, 41, and 44°C. At the end of the 90 min measurement period, the sensors were gently peeled off, and the skin was cleaned again. All tcpCO_2_ and *p*_*Se*_ *CO*_2_ data were recorded on computers for future analysis, and the room temperature was also recorded using a calibrated thermometer (Testo 735, Testo, Germany).

### 3.3 Data analysis

**FIGURE 5:**
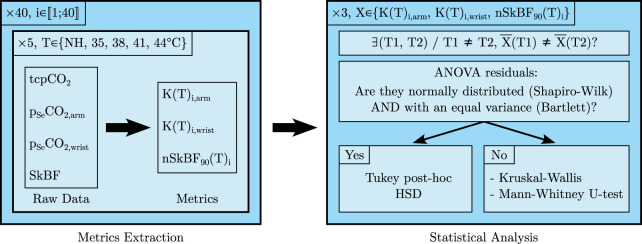
Graphical representation of the data analysis workflow, see the text for further details.

The data analysis workflow is summarised in Figure 5. Raw data were collected for all 40 subjects at five different temperatures and three metrics were extracted for each *i*th subject/temperature pair: *K* at the arm and wrist, and nSkBF_90_ at the arm only. For each of those metrics, an ANOVA was performed across all subjects to determine whether their mean values differ significantly between two temperatures. If the ANOVA residuals did not significantly differ from a normal distribution—according to Shapiro-Wilk testing—and if the hypothesis of variance equality between the temperature groups was also verified—according to Bartlett testing—a Tuckey *post hoc* HSD test was then performed. Otherwise, a Kruskal-Wallis test followed by a series of Mann-Whitney U-tests were performed. Additionally, Pearson and Spearman correlation tests were also carried out to study the influence of temperature on the three afore-mentioned metrics (not represented in Figure 5). When applicable, all tests were two-sided and a 5% alpha risk was chosen as significance threshold.

## 4 Results

### 4.1 Demographics and temperatures

The fourty subjects consisted in 24 men and 16 women, aged between 20 and 61 years (mean/median/Standard Deviation (SD): 40/39/13 years). The laboratory temperature was in the 20.1–22.7°C range for the whole duration of the experiments (mean/median/SD: 21.3/21.2/0.7°C). The skin temperature during the non-heated 18 min phase was measured twice, at 10 and 18 min after sensor application, and these two measurements were averaged to yield a single temperature value per subject. The latter was in the 27.1–31.8°C range (mean/median/SD: 29.3/29.2/1.2°C) at the arm, and in the 24.7–33.1°C range (mean/median/SD: 28.6/28.5/1.7°C) at the wrist.

### 4.2 Skin CO_2_ conductivity

**FIGURE 6:**
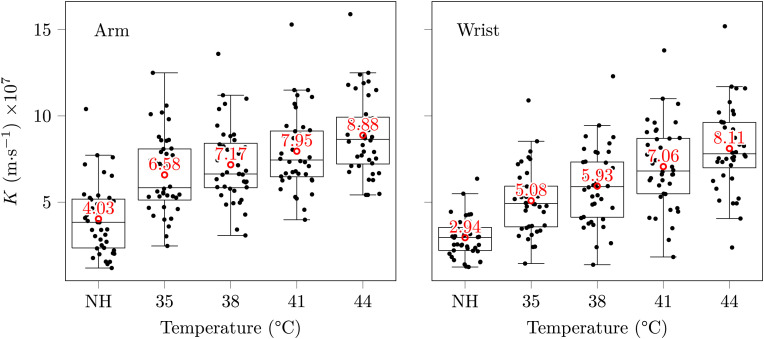
Skin conductivities at the arm and wrist. Each black mark corresponds to a subject/temperature pair, the red circles and texts indicate mean values, and some horizontal jitter was added to the black marks for legibility. The whiskers extend at most to 1.5 times the interquartile range, and descriptive statistics—range, and SD—are provided in Supplementary Material S1—two properties shared by the three box plots of the present paper.

The linear regressions leading to *K* values—see Section 3.1.1—yielded excellent regression coefficients, with average *R* ^2^ values of 0.98 and 0.96 at the arm and wrist, respectively. The resulting skin conductivities are summarised in Figure 6, and show a sharp tendency to increase with an increasing skin temperature. Interestingly, the five upper outlying values at the arm—at all temperatures—and the four upper and below outlying values at the wrist—in the 35–44°C range—belonged each time to a single subject, who exhibited an especially high, or low skin conductivity. However, these two latter subjects were not one and the same person at the arm and at the wrist. The dispersion of skin conductivity values is also glaring with max/min ratios for a given skin temperature/location pair in the 2.9–9.0 range.

A normalisation of the variable *K*—using the change of variable $K\mapsto \sqrt{K}$ —was carried out prior to the ANOVA, resulting in the Shapiro-Wilk and Bartlett tests to be passed (all p-values above 0.05). The ANOVA was significant (p-value below 10^–15^) and the results of the following Tukey *post hoc* HSD test are detailed in Table 1. The apparent increase in *K* with an increasing skin temperature seen in Figure 6 is hereby confirmed, with significantly different mean *K* values for most temperature differences except the closest ones—*i.e.* for the 35/38, 41/38 and 44/41°C pairs.

**TABLE 1:** p-values for the Tuckey HSD *post hoc* test for differences of the mean *K* at different temperatures.

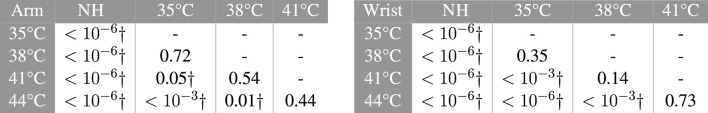

Values with a † are considered significant with a risk *α* = 0.05. (Only the lower-left part of the tables are filled-in for the sake of clarity).

Pearson and Spearman correlations were both significant (p-values below 10^–15^) with correlation coefficients of 0.60 and 0.59 at the arm, respectively, and 0.66 and 0.67 at the wrist. These coefficients indicate a moderate positive influence of the skin temperature on its diffusivity towards CO_2_.

### 4.3 Skin CO_2_ exhalation rate

In order to provide a more accessible parameter than *K* for the reader, as well as to allow direct comparison with existing literature, equivalent initial exhalation rates *Q* (*t* = 0) were also computed using Eq. 4. The resulting values are presented in Table 2.

**TABLE 2:** *Q* (*t* = 0) values, as computed using Eq. 4, expressed in cm^3^·m^−2^·h^−1^.

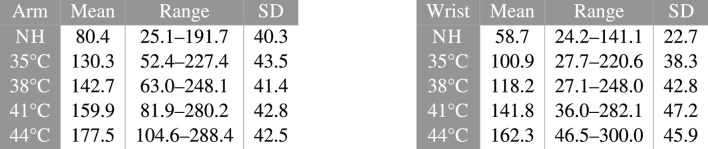

Although our results tend to indicate a higher CO_2_ exhalation rate at the upper arm than at the wrist—a MANOVA was performed considering the measurement site as independent variable and the five *Q*_*T*_ as dependent variables, and yielded a p-value of 0.01 using Pillai’s trace—the size of this effect is moderate, especially in view of the wide *Q* dispersion.

### 4.4 Laser Doppler Flowmetry

**FIGURE 7:**
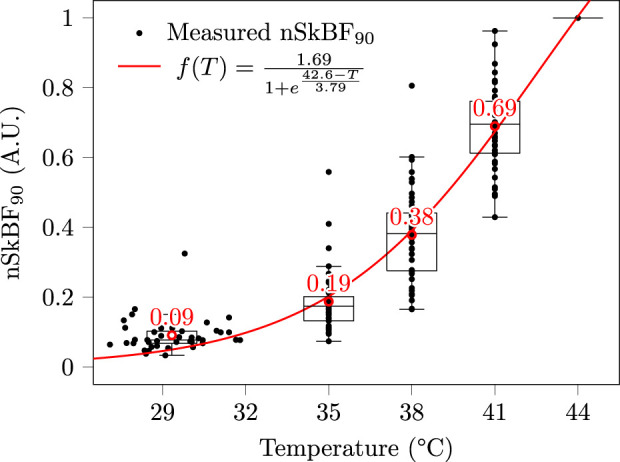
Measured nSkBF_90_ at the arm. Note that since nSkBF_90_ is normalised with respect to SkBF_90_ at 44°C, all subjects merge into a single unitary value at this latter temperature. Each black mark corresponds to a subject/temperature pair, the red circles and texts indicate mean values, and the red curve is a least square sigmoid fit. Contrary to Figure 6, no horizontal jitter was added to the data, and the dispersion observed in the 27–32°C range corresponds to the inter-subject variability in non-heated skin temperatures.

nSkBF_90_ values were computed as described in Section 3.1.2, and are presented in Figure 7. Skin perfusion exhibits a strong increase with temperature, with a tenfold multiplication between the NH basal state and the maximum vasodilation 44°C stage. In particular, a mild heating to a temperature of 38°C already entails a fourfold increase in perfusion. As was the case for skin conductivity, the dispersion of the nSkBF_90_ values is also considerable, with max/min ratios for a given temperature in the 2.2–9.7 range.

Regarding statistical analyses, the normality and variance homogeneity hypotheses could not be verified regardless of the changes of variable performed. A Kruskal-Wallis test was thus performed, followed by a series of Mann-Whitney U-tests, all of which proved significant (all p-values below 10^–10^).

Pearson and Spearman correlations were both significant (p-values below 10^–15^) with correlation coefficients of 0.90 and 0.96, respectively. These coefficients indicate a strong positive influence of the skin temperature on its perfusion. The fact that Pearson’s correlation is below Spearman’s is not surprising since the relationship between skin temperature and nSkBF_90_ is strongly non-linear, as emphasised by the sigmoid fit performed in Figure 7.

## 5 Discussion

The main objectives of this research were to ascertain the influence of skin temperature on *i)* its permeability towards CO_2_—through the study of the skin CO_2_ conductivity *K*, and exhalation rate *Q*—and *ii)* the skin blood flow—through nSkBF_90_.

### 5.1 Sensor design

#### 5.1.1 Skin contact surface

Although the sensor’s aluminium body was precisely machined following the drawing given in Supplementary Material S1, the exact surface area in contact with the skin that participates in gaseous exchange may slightly vary from one subject to another. Indeed, at each hole of the sensor’s sole, the skin forms a small dome, whose convexity is essentially function of the mechanical properties of the skin. Yet, those mechanical properties are sex-, moisture-, age-, and temperature-dependant (Salter et al., 1993; Held et al., 2018), thereby introducing small intra- or inter-subject variations in skin contact surface area. Since this area is used to calculate *K* through *S*_*Se*_—see Section 3.1.1—the latter is in turn influenced by these small variations.

While this would likely not change the conclusions of the present study given the order of magnitude of the above-described phenomenon reported in the literature, future research could look into replacing the grid-shaped sole that we used by a thin metal mesh, or a metallic foam. These two latter techniques were for instance implemented by Eletr et al. (1978), McIlroy et al. (1978), and Hansen et al. (1980). However, it must be emphasised that the shape of the sole of a thermally-regulated transcutaneous exhalation rate sensor is essentially a compromise between: *i)* the degree of perforation or porosity of the surface, which should be as high as possible to ensure a large diffusion surface, and *ii)* heat transfer considerations, which call for a plain, dense, surface, with a minimum number of holes, to ensure temperature homogeneity of the skin. Additionally, while the use of a wire mesh, or metallic foam reduces the above-mentioned “dome effect”, it also makes the surface estimation more tedious. Therefore, this aspect of the sensor design should be further investigated to find a satisfactory technical solution which addresses the above concerns.

#### 5.1.2 CO_2_ sensor choice

The choice of the selected NDIR CO_2_ sensor was mainly motivated by its compact form factor and ease of implementation. Additionally, the 5% range was especially adapted for CO_2_ diffusion rate measurement. Indeed, tcpCO_2_ in healthy subjects is typically in the 35–45 mmHg range (Rithalia et al., 1984), corresponding to 4.6–5.2% of CO_2_. Since the CO_2_ diffusion rate measurements taking place in the present study were only limited to the first moments of CO_2_ diffusion from the skin into the sensor—see Section 3.1.1—measured CO_2_ fraction values stayed below 1–2%. The 5% range was thus adapted to our need.

#### 5.1.3 Gas tightness

**FIGURE 8:**
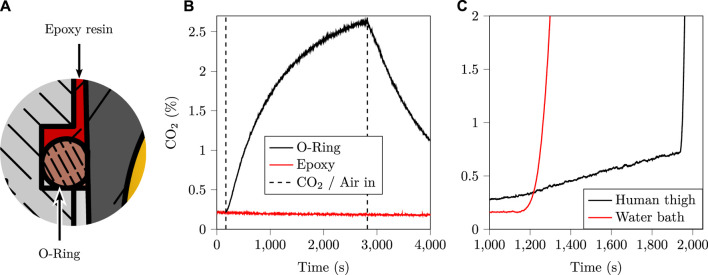
**(A)**: close up of the section view of Figure 2, showing the two key elements of the gas tight seal: a grease-coated O-ring, and cast epoxy resin. **(B)**: a sealing test comparing the greased O-ring alone, with the greased O-ring and the cast epoxy resin. This test clearly indicates that the greased O-ring alone does not accomplish gas tightness, whereas the cast epoxy resin does. **(C)**: sensor sensitivity towards humidity, showing the onset of condensation onto the gold-plated dome. This condensation drastically reduces the quantity of light reaching the detector, effectively blinding it, which is interpreted as an exceedingly elevated CO_2_ concentration.

As mentioned in Section 2.1, the gas tightness of the sensor’s chamber with respect to ambient air was of paramount importance for the success of the study. Indeed, any leak of inner-chamber CO_2_ towards the outer air would subtract from the measured rate of exhalation of CO_2_ through the skin, and thus impair the resulting *K* values. During the sensor’s design, gas tightness was assessed by sticking the sensor onto a glass plate using the same adhesive as for the human-testing part of the study. The so-obtained glass plate/sensor pair was then put inside a chamber which was successively filled with a 2.5% CO_2_/di-nitrogen (N_2_) mixture and fresh air. The resulting measurements are presented in Figure 8B, and clearly demonstrate that the greased O-ring alone was not gas tight, while the epoxy sealing was. The Figure 8A also illustrates in a cut view how the epoxy seal complements the O-ring. In practice, the grease-coated O-ring acts as a resin-proof sealing that prevents the resin from flowing inside the sensor’s chamber during its casting process.

This gas tightness allows CO_2_ to accumulate into the sensor chamber until an equilibrium is reached between the subcutaneous tissue and the sensor’s chamber—*i.e.* until *p*_*Se*_ *CO*_2_ = *tcpCO*_2_. While this equilibrium—although it would take several hours given the respective order of magnitudes of *Q* and *h*_*Se*_—and the associated CO_2_ diffusion process are at the very heart of this study, another undesirable chemical species will also accumulate into the sensor’s chamber due to the combined action of diffusion and sweating: water vapour.

While the influence of water vapour on NDIR CO_2_ measurements due to the infrared absorbance of water vapour is expected to be negligible given the large gap between CO_2_ and water vapour infrared absorption bands (Mranvick, 2023), the onset of condensation onto the reflective part of the sensor—namely the gold-coated reflective dome—can still be an issue. Indeed, the formation of micro-droplets of condensing water onto the latter dome would drastically reduce its reflectance, fooling the sensor into believing that a large amount of CO_2_ is present inside the sensor’s chamber—a well-known issue in NDIR sensing (Fietzek et al., 2014; Wang et al., 2018). In order to study the influence of condensing humidity levels onto the sensor used in the present research, two experiments were carried out whose outcomes are presented in Figure 8C. The first experiment consisted in placing the sensor on a human thigh at increasing temperatures and waiting for condensation to occur, which happened after 30 min at 44°C. The second experiment consisted in bubbling ambient air (20°C) through pumice stone inside a hot water bath, yielding water-saturated hot air (40°C), which was then flowed onto the un-heated sensor. Even in these unfavourable conditions—*i.e.* a cold sensor and water-saturated hot air—it took about 20 min to detect the onset of condensation on CO_2_ measurements. Given that the latter onset was particularly sudden and visible on *p*_*Se*_ *CO*_2_ in both experiments, the influence of water vapour condensation on this study was deemed negligible. Indeed, it would be easily detected—were it to happen while measuring a given subject—and the corresponding measurement would be discarded, something which did not happen in practice.

Finally, the reader should bear in mind that the gas tightness of the sensor and the accumulation of humidity underneath it both create a condition called *skin occlusion*. This occlusion, while out of the scope of this paper, has been studied by several authors (Frame et al., 1972; King et al., 1978; Faergemann et al., 1983), who reported much higher CO_2_ exhalation rates for long-term—*i.e.* days—occluded skin, as compared to its basal state. This phenomenon was not investigated in the present study due to the long time scale that it involves, but further research on this topic would be welcome.

#### 5.1.4 Sensors positioning

**FIGURE 9:**
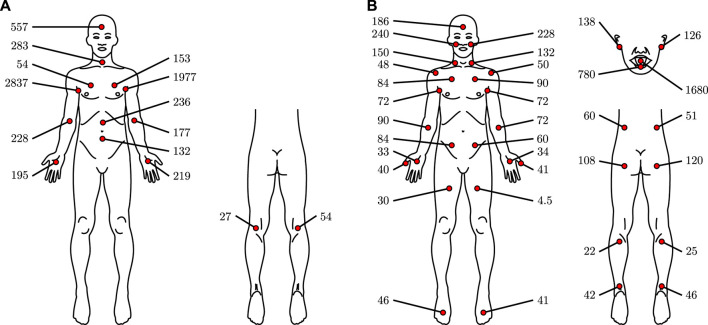
CO_2_ diffusion rates through human skin at various sites, expressed in cm^3^·m^−2^·h^−1^, based on data from Adamczyk et al. (1966) **(A)** and Levshankov et al. (1983) **(B)**.

To our knowledge, only three studies compared the influence of the measurement site on the transcutaneous CO_2_ diffusion rate in humans: that of Schulze (1943) on twelve subjects (Table 16 *op. cit.*), that of Adamczyk et al. (1966) on one subject and that of Levshankov et al. (1983) on an unspecified number of subjects. The results of the latter two authors are summarised in Figure 9—Schulze indications were difficult to interpret and were thus not illustrated.

The high variability in Adamczyk *et al.* data—probably caused by the inclusion of only one subject—is glaring, especially when studying left-body/right-body differences. Interesting are the extremely important values reported for the axilla. Those values may be measurement artefacts, or they may be caused by a peculiar behaviour towards CO_2_ diffusion of the apocrine glands, which are mainly located in the axilla—see Baker (2019). However, we found no evidence in the literature for or against this hypothesis. Alternatively, those elevated values may be caused by the skin temperature, which is much higher at the axilla on resting subjects than at the extremities (Niu et al., 2001; Sund-Levander et al., 2002), since the skin was not heated in their study. At the opposite, the results of Levshenkov *et al.* are more homogenous concerning left and right body measurements. All in all, and apart from the extreme axilla values, the reported CO_2_ diffusion rates exhibit no extreme variations and are of the same order of magnitude, regardless of the measurement site. In this aspect, it thus seems from the limited information at our disposal that no measurement site is far better than the other from the CO_2_ diffusion rate perspective.

Consequently, we chose our measurement sites mainly for their ease of access and acceptability, with a view to using these sites for a future wearable tcpCO_2_ sensor. In this respect, the dorsal side of the wrist and the upper arm were found to be particularly interesting, as evidenced by the rapidly expanding and widespread use of health-related wristband and armband in the recent years (Al-Eidan et al., 2018; Cosoli et al., 2020; Soon et al., 2020).

### 5.2 Skin CO_2_ conductivity

#### 5.2.1 Metric choice

It must be emphasised here that in the simplified skin diffusion model introduced in our previous publication (Dervieux et al., 2022) and detailed in Figure 3, the membrane called “skin” does *not* correspond to an actual physiological membrane. Consequently, its thickness *w* and diffusivity towards CO_2_ does *not* correspond to any physical property that might have been measured on a specific part of the dermis or epidermis. Rather, this “skin” membrane corresponds to a physical modelling of gas transport between the subcutaneous tissue and the outer air. As such, the latter membrane models both the diffusion of CO_2_ through the stratum corneum *and* the circulation of blood and diffusion of CO_2_ in the dermis and subcutaneous tissues.

Moreover, this model also integrates the difference in tcpCO_2_ between that measured by the reference Radiometer tcpCO_2_ monitor, and that measured at the sensor’s location. Indeed, since the reference tcpCO_2_ monitor was set to 41°C, it is likely that the tcpCO_2_ that we injected in Equation 4 is slightly over-estimated—as per the dilution principle presented in Figure 11—at temperatures below 41°C. Consequently, reported *K*—or *Q*—values below 41°C are likely to be slightly over-estimated. The amplitude of this over-estimation should be in the same order of magnitude as the arterio-venous pCO_2_ gradient in resting, healthy subjects—*i.e.* about 5–15% in the NH–38°C skin temperature range (Kowalchuk et al., 1988; Schneider et al., 2013). However, this state of fact was inevitable since, to the best of our knowledge, no clinical tcpCO_2_ monitor working at a temperature below 37°C exists at the time being, and manufacturers recommend using 41–42°C—an injunction that we followed. Future research aiming at extending our work may consider the design of a tcpCO_2_ sensor working at low temperature in order to establish the appropriate corrections to the obtained *K* values.

#### 5.2.2 Impact on the response time of a future tcpCO_2_ sensor

Contrary to perfusion—which increases over elevenfold with skin heating—*K* only doubles from NH to 44°C, and its increase is even smaller between 35–38 and 44°C values. This latter fact is all the more interesting when having in mind the design of a future energy-efficient tcpCO_2_ sensor. Indeed, internal studies measuring skin temperature under a wearable device positioned at the upper arm (Bora Band, Biosency, France) on ten healthy subjects revealed that a mean skin temperature of 33.9°C could easily be achieved at the upper arm without additional heating, and that covering the arm with an additional layer of isolation—*i.e.* shirt or jumper sleeves—makes it rise even higher to reach 35.1°C.

With such skin temperatures, there is no strong incentive—from a response time point of view—to heat the skin actively any further—*i.e.* by mean of an external electrical heating system. Indeed, the measured increase of 35% in *K* at the arm from 35 to 44°C—see Figure 6—would result in a decrease in response time of the same magnitude for a given tcpCO_2_ sensor, according to the response time model presented in our previous paper (Dervieux et al., 2022). While having a slower sensor may seem like a burning issue for critical care applications, it is not the case for telemonitoring for which long-term tendencies are to be observed over several months (Jang et al., 2021).

Additionally, it should be noted that since the *Q* values measured in the present study—80–178 cm^3^·m^−2^·h^−1^ on average—are in line with that used in our previous publication (Dervieux et al., 2022) for response time calculations—100 cm^3^·m^−2^·h^−1^—the afore-proposed sensor thickness of 100 μm for a response time below 10 min remains credible. As a reminder, it was shown in the latter publication that a linear relationship exists between the response time of an equilibrium-based tcpCO_2_ sensor, and the volume to surface ratio—*i.e.* equivalent thickness—of its equilibration medium. Thus, there is essentially a compromise to be made between this thickness, which cannot be infinitely small for technological reasons, and the response time of a so-designed sensor (Dervieux et al., 2022).

Of note, and to the best of our knowledge, there is a lack of clinical guidelines specifying the required response time for tcpCO_2_ monitors. Nevertheless, there exists a considerable amount of literature focusing on transcutaneous monitor testing in clinical environments, from which it can be inferred that a typical *in vitro* 90% response time of about 1 min (Bendjelid et al., 2005; Eberhard, 2007) is achievable with current tcpCO_2_ monitors. *In vivo* performance reports, on their, part mention an approximatively 10 min initial equilibration time before a first tcpCO_2_ reading can be taken (Carter and Banham, 2000; Domingo et al., 2010; Restrepo et al., 2012). Regarding the response time of tcpCO_2_ monitors following a sudden change in paCO_2_, a lag has been reported in the literature between end-tidal pCO_2_—petCO_2_—paCO_2_, and tcpCO_2_, inducing a higher *in vivo* response time than in the ideal *in vitro* case. Reported values for this latter lag fall within the 1–5 min range (Kesten et al., 1991; Carter and Banham, 2000; Cuvelier et al., 2005; Rafl et al., 2018). An overall response time requirement of approximatively 5 min can thus *de facto* be assumed for a tcpCO_2_ monitor to meet field expectations. Still, this latter assumption mainly holds for the intensive care of critically ill patients (Mari et al., 2019) and no information exists concerning long-term tcpCO_2_ (tele-)monitoring for the obvious reason that the corresponding monitors do not exist yet.

### 5.3 Exhalation Rate

#### 5.3.1 An Imperfect Metric

**FIGURE 10:**
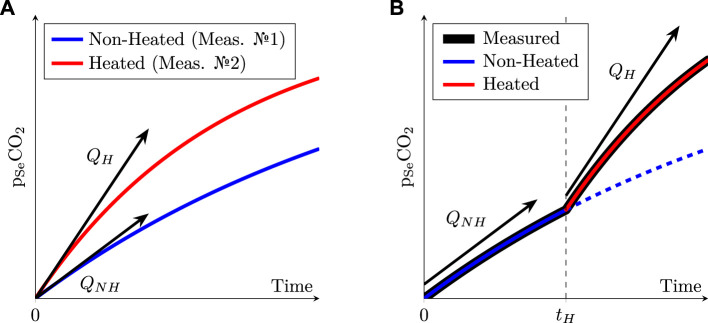
**(A)**: schematic evolution of the pCO_2_ inside the sensor’s chamber when the skin is heated or not. **(B)**: same as Left, but with a non-heated sensor placed onto the skin for a duration *t*_*H*_ before being heated. Note the difference between the two sensing schemes: the left one requires two successive measurements—one heated, the other one non-heated—while the right one consists in a single measurement during which the skin is successively non-heated and then heated. The dashed line on the right represents what would have happened without heating during the whole acquisition, which is equivalent to the solid blue line on the left.

Considering Equation 1, it readily appears that the exhalation rate *Q* is not constant, and logically depends on the initial *p*_*Se*_ *CO*_2_, and of the passing of time. This issue has however been largely ignored by the literature on the topic—see Table 3—and *Q* has been considered by most authors as if it had a single constant value. The latter, which has been reported as *the* CO_2_ diffusion rate through the skin, is actually the *initial one in free air*—*i.e.* Q (t=0) with *p*_*Se*_ *CO*_2_(*t* = 0) ≈ 0—and corresponds to the slopes of the tangents to the *p*_*Se*_ *CO*_2_ curves at *t* = 0 in Figure 10A.

**TABLE 3:** CO_2_ exhalation rates through the skin in the literature compared with the present studies.

Exhalation rate *Q* [cm^3^·m^−2^·h^−1^]	Temp. [°C]	Num. Of Subjects	Reference
25–120	22–36 (air)	2	Shaw and Messer (1930a)
10–160	25–37 (air)	1	Shaw and Messer (1930b)
**58–169**	**26–31 (air)**	**38**	**Ernstene and Volk (1932), Table 1**
12–143	23–37 (air)	1	Whitehouse et al. (1932)
**32–69**	**25–28 (air)**	**13**	**Schulze (1943), Table 12**
180–2500^†^	–	1	Adamczyk et al. (1966)
25–87	–	5	Thiele and Van Kempen (1972)
11–28	25–35 (air)	3	Frame et al. (1972)
**50**	**–**	**27**	**Levshankov et al. (1983)**
**140–221**	**36 (skin)**	**14**	**Eöry (1984)**
25–192	27–32 (skin)	40	This work, NH arm
24–141	25–33 (skin)	40	This work, NH wrist
25–288	27–44 (skin)	40	This work, all temp. arm
24–300	25–44 (skin)	40	This work, all temp. wrist

Past studies with more than ten participants are indicated **in bold** and were used for sample size determination.

^†^ Axilla measured value, possibly erroneous.

Unfortunately, if measuring *Q* as illustrated in the latter figure is theoretically feasible at different temperatures, it would also require to remove the sensor at each temperature change, in order to renew the gas inside the inner chamber of the sensor with fresh air. This would in turn require to peel off the sensor from the subject’s skin at each temperature change, which would distort the *Q* measurement, as the skin—and more specifically the *stratum corneum*, its outermost layer—would become thinner and thinner at each sensor replacement—actually, stripping the skin with multiple tape applications is a well-known technique to drastically increase *Q* (Scheuplein, 1976; Eletr et al., 1978; Greenspan et al., 1981).

Thus, the sensor was left in place in this study, while the temperature was successively changed from NH up to 35, 38, 41, and finally 44°C. This led to measured *p*_*Se*_ *CO*_2_ alike that represented in Figure 10B. In that case, using *Q* as a metric would be unpractical, since the *p*_*Se*_ *CO*_2_ value at *t*_*H*_ is not null, and *Q* values would no longer represent *initial* CO_2_ diffusion rates as measured in free air. In practice, the obtained *Q* values at different temperatures would then not be comparable with each other, each one being measured with a slightly different *p*_*Se*_ *CO*_2_ initial value.

#### 5.3.2 Comparison with existing literature

Thanks to Equation 4, we can however obtain equivalent *Q* values in free air from our *K* measurements, and compare them with those of the literature, given in Table 3—at least for the NH case. The values reported in Tables 2 and 3 are of the same magnitude, and the wide amplitudes that we report here—*e.g.* 25–192 and 24–141 cm^3^·m^−2^·h^−1^ at the NH arm and wrist, respectively—are on par with those reported in previous research.

### 5.4 Laser doppler flowmetry

#### 5.4.1 Choosing nSkBF_90_ as a metric

Both inter-subject and inter-site LDF variabilities have often been reported in the literature (Johnson et al., 1984; Cracowski et al., 2006; Minson, 2010; Roustit and Cracowski, 2012; Cracowski and Roustit, 2016; Hodges et al., 2016), and appears to be inherent to this modality of skin blood flow measurement as well as to human physiology in general. Nonetheless, certain guidelines may be followed to obtain the most reproducible results (Cracowski et al., 2006). In particular, when it comes to derive a single explicit LDF metric from a given measurement period—such as a skin-site/sensor-temperature pair, for instance—several techniques have been developed to obtain meaningful results from raw LDF data.

At first, some authors—*e.g.* Hodges et al. (2016)—prefer to express the skin blood flow as Cutaneous Vascular Conductance (CVC), which is given by the LDF in P.U. or V, divided by the Mean Arterial Pressure (MAP). The CVC is said to be more “physiological” (Cracowski et al., 2006), since an increase in skin blood flow could be caused by an increase in MAP but also by an increase in vascular compliance, for instance. By dividing the LDF-acquired blood flow by the MAP, the obtained CVC value is thus in theory more representative of the arteriovenous compliance, a theory supported by several works in haemodynamics (Johnson, 1986; Lautt, 1989; Herring and Paterson, 2018). At the same time, skin blood flow alone—often abbreviated as SkBF, and either expressed in P.U. or Volts—has been used for several decades (Johnson et al., 1984; Frantz et al., 2012) and remains a good alternative to CVC when MAP is not available.

Then, once the type of measurement—SkBF or CVC—is chosen, the question that comes next is that of the extraction of a single perfusion metric from a long-lasting acquisition. Indeed, due to the peculiar dynamics of thermal hyperaemia—see Figure 4, above—a simple time-averaging on the whole acquisition duration would make little sense.

To circumvent this issue, several research teams used temporal averaging on manually-set periods of interest in the raw LDF data. The averaging duration that they used depended on the studied phenomenon, with durations of 1–3 min for transient phenomena—*i.e.* initial bump and after-bump nadir—up to 5–10 min for long-lasting ones—*i.e.* baseline or maximum perfusion plateau (Minson et al., 2001; Frantz et al., 2012). Other authors, for their parts, chose to average the two to three last minutes of a 10–25 min measurement window at the maximal perfusion value, which obtention is detailed below (Kellogg et al., 2008; Hodges et al., 2016). However, Barcroft and Edholm (1943) and Taylor et al. (1984) mentioned even longer durations for thermal hyperaemia to fully settle following a change in skin temperature—up to 40–60 min. Such a lengthy onset period would result in a total acquisition duration in the 3–5 h range for the five different temperatures involved in the present study. At the opposite, a total experiment duration—including informing the subjects and obtaining their consent—of about 2 h seemed to us to be an acceptable maximum for easily recruiting volunteers. This 2 h duration in turn entails that each temperature window of the present study only lasted 18 min, which may not be enough for the establishment of the nitric-oxide mediated hyperaemia detailed in Figure 4. Thankfully, this 18 min duration is by far long enough for the axon mediated response to take place, and the latter often yields perfusion levels comparable to that reached at the end of the nitric-oxide mediated phase (Kellogg et al., 2008; Minson, 2010; Frantz et al., 2012). Thus, by taking the 90-th percentile of SkBF—see Section 3.1.2—the obtained SkBF_90_ values are likely to be representative of the SkBF plateau values which would have been observed by increasing the duration of the temperature windows. The latter hypothesis is further confirmed by the similarity between our results and that of the literature, as discussed in the next section.

Finally, it is also common practice to normalise the measured skin blood flow—whether expressed as SkBF or CVC—by its maximum value, often taken after a prolonged (≥15 min) period at an elevated (≥44°C) temperature (Taylor et al., 1984; Vionnet et al., 2014; Hodges et al., 2016) or by direct injection of sodium nitroprusside (Kellogg et al., 2008). Although it has been seldom proposed to normalise the measured values by the baseline blood flow value instead of the maximum one (Magerl and Treede, 1996; Mayrovitz and Leedham, 2001), this is considered bad practice because intra-subject baseline variations can be important even in a temperature controlled room (Bircher et al., 1994; Cracowski et al., 2006).

In this study, CVC was not considered due to the invasiveness of a continuous MAP measurement and SkBF was thus chosen as raw perfusion metric. Then, we proposed to take the 90-th percentile of a given temperature window instead of time averaging. Finally, normalisation by the maximum perfusion value—*i.e.* the one reached at the end of the 44°C window—was performed, as per literature guidelines.

#### 5.4.2 Comparison with the literature

The LDF measurements that were gathered in the present study are consistent with existing literature on the topic. In particular, the sigmoid behaviour observed in Figure 7—revealing a strong onset of hyperaemia in the 35–41°C range—is on par with the observations of Magerl and Treede (1996), Stephens et al. (2001), and Hodges et al. (2016).

#### 5.4.3 Impact on the accuracy of a future tcpCO_2_ sensor

**FIGURE 11:**
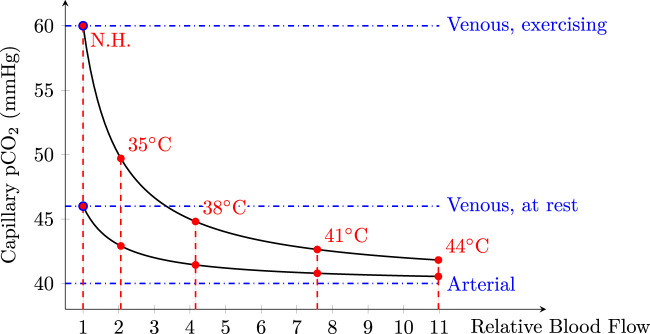
Capillary pCO_2_ as a function of relative blood flow considering two venous pCO_2_ levels: at rest, and while exercising. Relative blood flow values measured in the present study were also added in red with their respective temperature labels. A normal paCO_2_ of 40 mmHg (Schneider et al., 2013) was taken for arterial blood, while venous blood levels were set to 46 and 60 mmHg at rest and while exercising, respectively. Of note, while 46 mmHg at rest is generally accepted in the literature (Byrne et al., 2014), the 60 mmHg exercising value was mainly chosen for legibility reasons. Indeed, exercising values may exceed 100 mmHg during heavy exercise, or in case of septic shock (Kowalchuk et al., 1988; Diaztagle Fernández et al., 2017). Modified from Rooth et al. (1987).

The fact that the perfusion is doubled at 35°C and quadrupled at 38°C compared to baseline—see Figure 7—is especially encouraging for the development of a future energy-efficient tcpCO_2_ sensor, since these temperatures can be easily achieved without—or with minimal—heating, as already discussed in Section 5.2.2 (reaching over 35°C at the upper arm under jumper sleeves). Indeed, since arterialised capillary blood—either obtained by local heating or application of a vasoactive cream—is gaseously close to arterial blood (Zavorsky et al., 2007), it is to be expected that partially arterialised capillary blood obtained by a mild heating—*i.e.* below 44°C—lies somewhere between venous and arterial blood, from a gaseous content point of view. More specifically, Rooth et al. (1987) hypothesised that the subcutaneous capillary pCO_2_—*i.e.* tcpCO_2_—would be a barycentre between venous and arterial pCO_2_, as illustrated in Figure 11.

This figure emphasises the fact that—especially for a resting subject—even a mild heating of the skin in the 35–38°C range could be enough to yield a tcpCO_2_ only a few mmHg away from the paCO_2_. The latter error may be acceptable depending on the clinical application targeted. For example, the Food and Drug Administration (FDA) requires tcpCO_2_ monitors to be accurate within 5 mmHg, with an allowed drift of up to 10% of the initial reading over a 1-h period (Food and Drug Administration, 2002).

### 5.5 Sample size

The main objective of the present study was to estimate the mean *K* value as a function of temperature. The latter mean can be estimated at each temperature *T* by:

$${\hat{K}}_{T}=\frac{1}{N}\cdot \overset{N}{\underset{i=1}{\sum }}K_{S,i}$$ (7)

wherein the index *i* stands for the *i*th subject of the study and *N* stands for its sample size. Contrary to hypothesis testing, for which a sample size may be derived straightforwardly from targeted alpha or beta risks, and some prior knowledge of the data (Ambrosius, 2007, Chap. 19; Chow et al., 2017), sample size determination in the case of an exploratory—or pilot—study is more challenging, with its share of arbitrary decision (Ko and Lim, 2021). Indeed, while a 95% confidence interval can be computed for *K*_*T*_ as:

$$C.I._{K_{T}}^{95\%}=\left[{\hat{K}}_{T}-\varepsilon ,{\hat{K}}_{T}+\varepsilon \right],\;\text{and}\;\varepsilon =-t_{N-1}\left(\frac{0.05}{2}\right)\cdot \frac{s}{\sqrt{N}}$$ (8)

wherein *t*_*N*−1_ is the percentile score of a Student distribution with *N* − 1 degrees of freedom, and *s* is the SD of the sample—*i.e.* the *estimated* standard deviation of the population—the value of the latter SD is vastly unknown. In order to estimate an adequate sample size for the study at hand—based on an *acceptable* margin of error on ${\hat{K}}_{T}$ —a prior estimation of *s* is thus needed, and is the object of the upcoming section. Importantly, since *Q*—that is *Q* (*t* = 0) using the above-presented notation—was studied by earlier authors instead of *K*, the following reasoning will be made using the former. This can be done safely since the two values are linked by a proportionality constant—see Equation 4. Of note, Equation 8 holds only if the *K*_*T*_ follow a normal distribution, which had neither been confirmed nor denied in the literature, to the best of our knowledge, but which was verified in the current study—see Section 5.5.3.

#### 5.5.1 Literature review

Among the literature studies on the topic of skin CO_2_ exhalation rate measurements on human subjects detailed in Table 3, only four of them have been performed on more than ten subjects, and are highlighted **in bold** in the aforementioned table. Unfortunately, the latter studies are sometimes unclear about *Q* measuring conditions—measurement site and skin temperature, in particular. We did our best not to distort or misinterpret the works of their authors, but what follows is essentially our best *interpretation* of their writings. These four studies reported—or made it possible to derive from raw data—a $\hat{Q}$ and *s* value for each measurement site investigated. These data can be used to compute 95% confidence intervals on *Q* estimation—as shown in Equation 8—or on *s*, yielding (Ambrosius, 2007, Chap. 4):

$$C.I._{\sigma }^{95\%}=\left[\frac{s^{2}\cdot \left(N-1\right)}{\chi_{N-1}^{2}\left(\frac{0.05}{2}\right)}\;;\;\frac{s^{2}\cdot \left(N-1\right)}{\chi_{N-1}^{2}\left(1-\frac{0.05}{2}\right)}\right]$$ (9)

wherein $\chi_{N-1}^{2}$ is the percentile score of a *χ* ^2^ distribution with *N* − 1 degrees of freedom. The resulting confidence intervals are reported in Table 4 and tend to indicate a relative uncertainty on *Q* estimation in the order of 5–30% for relatively small sample sizes—*i.e.* 13–38 subjects.

**TABLE 4:** Confidence intervals at the 95% level for *σ* and *Q*, computed from *s* and $\hat{Q}$ values reported in the literature. Aside from the relative uncertainties—defined as 2 ⋅*ɛ*/*X* wherein *X* is *σ* or *Q*—all values are given in cm^3^·m^−2^·h^−1^.

	Lower bound	Reported	Upper bound	Relative uncertainty (%)	Measurement site	Reference
*σ*	18.7	22.9	29.6	48	Whole arm	Ernstene and Volk (1932)
	8.5	11.9	19.6	93	Abdomen	Schulze (1943)
	2.65	3.36	4.60	58	Left forearm	Levshankov et al. (1983)
	2.41	3.06	4.19		Right forearm	Levshankov et al. (1983)
	15	21	34	89	Acupuncture site	Eöry (1984)
	13	18	28		“adjacent skin area”	Eöry (1984)
*Q*	113	120	128	13	Whole arm	Ernstene and Volk (1932)
	41	48	55	30	Abdomen	Schulze (1943)
	48.0	49.3	50.7	5.39	Left forearm	Levshankov et al. (1983)
	48.4	49.6	50.8	4.88	Right forearm	Levshankov et al. (1983)
	209	221	233	11	Acupuncture site	Eöry (1984)
	130	140	150	14	“adjacent skin area”	Eöry (1984)

#### 5.5.2 Chosen sample size

The reported *s* value, as well as the upper and lower bounds of *s* 95% confidence interval were then used to compute the relative uncertainty on *Q* as a function of the number of subjects using Equation 8. While this relative uncertainty decreases when the sample size increases, its reducing rate—as well as the associated uncertainty values—varies wildly depending on the considered data source. Indeed, while a sample size of 20–30 subjects should lead to a relative uncertainty on *Q* in the 5–10% range using Levshankov et al. (1983) data, much larger sample sizes—*i.e.* 100-150 subjects—would be needed to reach the same level of accuracy using Schulze (1943) or Ernstene and Volk (1932) *.* measurements. In the end, since the works of the latter two authors were much older—1932 and 1943, respectively—than that of Eöry (1984), Levshankov et al. (1983) and , respectively—it was decided to put them aside. The sample size determination was thus grounded only on the works of Eöry (1984), Levshankov et al. (1983), and a sample size of 40 subjects was deemed acceptable, since it should have resulted into a relative uncertainty on *Q* estimation below 10%.

#### 5.5.3 Results

Unfortunately, this initial estimation of a 40 subjects cohort proved to be rather optimistic in practice. Indeed, the relative uncertainty on measured *Q* values can be computed using Equation 8, and falls in the 15–32% range, depending on the skin temperature and measurement site. In this aspect, our results are close to those presented by Ernstene and Volk (1932), Schulze (1943), and Eöry (1984) who reported relative uncertainties in the 11–30% range. Yet, the latter authors used less than 40 subjects, and our uncertainty range was thus expected to be narrower than theirs. Moreover, the present study also exhibits a higher variability than that of Levshankov et al. (1983)—whose results indicate a relative uncertainty about 5%, see Table 4.

The origin of these discrepancies between literature-driven expectations and the above-presented results is not fully understood at the moment. One possible explanation could be differing measurement sites between the above-mentioned studies and the ones that we chose. Indeed, the data reported by Eöry (1984), Schulze (1943) (Table 16 *op. cit.*) tend to indicate some degree of variability in the relative uncertainty expected at different sites—in particular when comparing the abdomen and hand in Schulze’s data (30 vs. 45%), or the two sites used by Eöry (10 vs. 14%). Thus, it seems plausible that *Q* variability at the upper arm and wrist is above that reported in earlier studies for differing sites.

Ultimately, we cannot but recommend using larger sample sizes in future studies of a similar nature, considering the significant variability we observed. As a side note related to sample size determination, the normality of the *Q* distribution was ascertained using a series of Bonferroni-corrected Shapiro-Wilk tests which were non-significant, further justifying the approach presented in Section 5.5. To the best of our knowledge, this is the first report of normality for transcutaneous CO_2_ exhalation rates.

## 6 Conclusion

As stated in introduction, the aim of this study was twofold: measuring the influence of skin temperature on the transcutaneous diffusion of CO_2_, and on the skin blood flow. To this end, a custom sensor was designed and used on 40 healthy human subjects at two measurements sites: the upper arm, and the wrist.

Our results indicate comparable behaviours at both sites, with an increasing relationship between temperature on the one hand, and CO_2_ exhalation rate, CO_2_ conductivity and perfusion on the other hand. These results are encouraging for the development of a future energy-efficient tcpCO_2_ sensor for the following reasons:

– Skin conductivity towards CO_2_ increases only moderately with an increase in skin temperature, at most doubling from NH to 44°C. Thus, if the response time of the sensor-to-be is not critical—*i.e.* if a 35% slower response is acceptable compared to the one reachable at maximum skin heating—the latter may not require additional heating. This is especially encouraging in the perspective of building a wearable, battery-operated device.
– Perfusion, for its part, increases strongly with an increase in skin temperature, already doubling from NH to 35°C, and quadrupling from NH to 38°C. This phenomenon is especially interesting since—according to Rooth et al. (1987)—this should bring tcpCO_2_ close to paCO_2_ even for skin temperatures as low as 35–38°C, which are reachable at the arm without additional heating, given that the latter is covered by warm clothings. However, this latter hypothesis—*i.e.* the existence of a clinically-satisfying tcpCO_2_/paCO_2_ correlation in the 35–38°C skin temperature range—is yet to be demonstrated experimentally *in vivo*, which will be the subject of future research.

Additionally, our results highlight the significant variability of transcutaneous CO_2_ exhalation rate and conductivity measurements in human subjects. Hence, we strongly advise future research on the topic to consider large sample sizes—*i.e.* more than 40 subjects—in order to ensure accurate estimates of the latter metrics. The present study also focuses only on two measurement sites—the upper arm and the wrist—and further investigations at other sites would be welcome. In particular, the remarkably high axilla values reported by some authors is intriguing, and could benefit from a special attention. Of note, the study data—demographics, *K*, *Q*, and nSkBF_90_ values—are provided in Supplementary Material S2.

## Data Availability

The original contributions presented in the study are included in the article/Supplementary Material, further inquiries can be directed to the corresponding author.

## Footnotes

1 Typical value for a TCM4 tcpCO_2_ monitor (Radiometer, Denmark), as measured by the authors.
