# Supplementary material for: Skin temperature influence on transcutaneous carbon dioxide (CO2) conductivity and skin blood flow in healthy human subjects at the arm and wrist
Source: Front Physiol. 2024 Jan 23;14:1293752. doi: 10.3389/fphys.2023.1293752 (PMC10846589; doi:10.3389/fphys.2023.1293752)
Supplement: Supplementary file 1 [file DataSheet1.PDF]

Supplementary Material S1

—

Technical Information

1 Sensor Design

1.1 Mechanical Drawings

Complete mechanical drawings of the sensor's aluminium body are given at the end of the present document, in page 6. Aluminium was chosen for its high thermal conductivity—134 $W.m^{-1}.K^{-1}$—as well as its low density—2.79[1]. The choice of pure copper was also considered, as it has a higher thermal conductivity than aluminium—401 $W.m^{-1}.K^{-1}$—but it is alas also much denser—with a density of 8.9[2]. As it seemed desirable to make the sensor as lightweight as possible in order to minimise skin blood flow perturbations, aluminium was ultimately chosen. Indeed, several works indicate that even a mild—*i.e.* above 10 mmHg / 1.3 kPa—local cutaneous pressure can significantly impact local blood flow[3–5]. Besides, aluminium does not oxidise as copper does, and can be considered safe for skin contact, as further discussed in Section 1.4.2.

1.2 Power and Supply Block

The power and supply block is illustrated in Figure 1, Left. It consists in:

- an interfacing Printed Circuit Board (PCB) similar to that used on top of the sensor, which enables connection with the latter by mean of an eight-pins microfit Molex cable,
- a thermostat board (XH-W1209, Shenzhen Global Technology, China) allowing for the temperature regulation of the sensor's aluminium body,
- a Universal Serial Bus (USB) to Universal Asynchronous Receiver Transmitter (UART) converter (CP2102, Silicon Labs, USA), which enables connection between the carbon dioxide (CO_2) sensor—through UART—and the computer—through USB,
- a 230 V-AC to 12 V-DC / 10 A switching power supply onto which the afore-mentioned elements are affixed by mean of a 3D-printed contraption (black plate in Figure 1),
- a 3D-printed cover (not shown) that prevents contact with any exposed electrical conductor, while allowing the USB and Molex cables to be plugged, and the temperature to be adjusted.

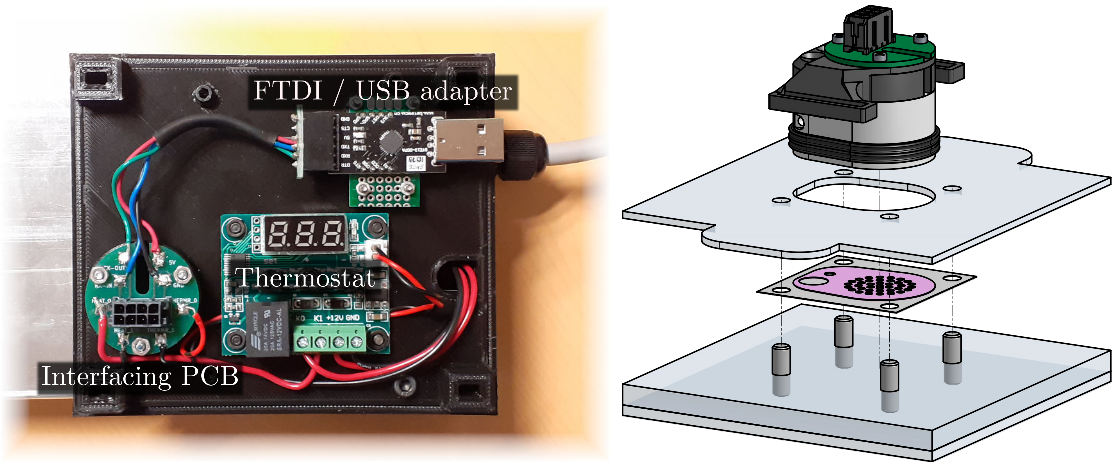

Figure 1: **Left:** the power and supply block. **Right:** the adhesive mounting jig with—from top to bottom—the sensor, the centring layer, the adhesive stuck onto its release liner, and the chassis.

1.3 Adhesive Tooling

A custom mounting jig was developed to ensure an accurate and reproducible positioning of the laser-cut, double-sided, clinical-grade tape (1567, 3M, USA) onto the sensor's sole. It is represented in Figure 1, Right, and allows the user to: ① put a release liner—with the adhesive stuck on it—onto a chassis, positioning it by means of the four steel studs integrated into the latter, ② add a centring layer above the liner, and ③ stick the sensor onto the adhesive, with the centring of the former being ensured by an appropriate cut made into the centring layer. Finally, when ④ the user removes the sensor, the adhesive remains stuck onto it, precisely aligned, and the release liner remains onto the chassis. The sensor is then ready to be applied on a subject's skin.

1.4 Safety Issues

Three distinct safety concerns have been identified regarding the application of the sensor on the skin of human subjects: thermal injury hazard, dermatological reaction hazard, and electric shock hazard.

1.4.1 Thermal Injury Hazard

Given the relatively low thermal power of the heating wire (6.1 W), the temperature of the sensor cannot reach excessive values within minutes, even in case of thermostat malfunction. Would the latter occur, both the investigator and the subject would notice it thanks to the external reference thermometer, and cutaneous sensation, respectively. In addition, the enamelled resistive wire is covered by two quad rings, guaranteeing that neither the subject nor the investigator may touch it directly during the experimentations.

The only relevant source of thermal injury is thus long term burn. Literature on the topic suggests a maximum long-term skin temperature of 42°C, but higher temperatures can be used for a limited amount of time—up to 45°C for a few minutes for instance[6], or up to several hours depending on the study[7]. In the medical practice of tcpCO_2 monitoring, temperatures

up to 45°C were used for up to one hour[8] or 42°C for eight hours[9]. Temperatures in the 40–44°C are also often encountered, since temperatures above 42°C yield the most accurate $tcpCO_2$ measurements[10]. Legally speaking, the Food and Drug Administration (FDA) guidance for $tcpCO_2$ monitors recommends not to exceed 44°C for more than four hours[11]. Consequently, since the sensor will not be used for more than 90 min on each subject, a highest temperature set-point of 44°C was deemed safe.

1.4.2 Dermatological Reaction Hazard

The body of the sensor is made out of solid aluminium, which is vastly inert towards the skin. Indeed, most allergic reactions related to aluminium come from aluminium ions used in vaccines and not from solid metal[12, 13]. Although some rare case of reaction to thin solid aluminium particles have been reported, they concern factory workers being exposed daily to aluminium dust for months—if not years—prior to sensitisation[14, 15]. Thus, it was considered most unlikely that the subjects exhibit any reaction to the aluminium sensor's body.

Then, the other parts of the sensor that may be in close contact with the skin are the Doppler probe and double-sided adhesive. Since both the latter were designed for skin application and are medical-grade products, any adversarial reaction with the skin is also highly unlikely to happen.

Finally, for hygienic reasons, the sensor was also cleaned with a disposable paper towel soaked in a 70% isopropyl alcohol / 30% water mixture prior to measurements, which is standard medical practice for disinfection.

1.4.3 Electric Shock Hazard

The supply block is designed so that no bare high voltage conductor is exposed. Additionally, all the electrical connections to and from the sensor itself are only raised to low voltage—12 V at most—ensuring that no electric shock can possibly happen, even if the insulation of one or several wires were to fail.

2 $tcpCO_2$ Extraction

The subject's capnia was monitored using a clinical grade transcutaneous capnometer (TCM4, Radiometer, Denmark), which recorded $tcpCO_2$ at a sampling rate of 1 min^{-1}. In order to get only one $tcpCO_2$ value per temperature window—as required for K calculations—the raw $tcpCO_2$ signal was processed as follows:

- the first ten minutes of the acquisition were truncated to allow the sensor to stabilise,
- the remaining acquisition was split in five segments, each segment corresponding to a temperature window—Non-Heated (NH), 35, 38, 41 and 44°C,
- for each segment, the measured $tcpCO_2$ was then averaged to yield a single value, which was then used to compute K, as described in the companion paper.

This approach was deemed appropriate since $tcpCO_2$ in healthy resting subjects was fairly stable (mean / median standard deviation on all acquisitions: 0.92 / 0.78 mmHg).

3 Box-Plot Descriptive Statistics

Mean, ranges, and SD of K and $nSkBF_{90}$ measurements are given in Table 1 and 2, respectively.

Arm	Mean	Range	S.D.
N.H.	4.03	1.18–10.40	2.03
35°C	6.58	2.46–12.50	2.26
38°C	7.17	3.07–13.60	2.19
41°C	7.95	3.98–15.30	2.24
44°C	8.88	5.42–15.90	2.28

Wrist	Mean	Range	S.D.
N.H.	2.94	1.24–6.36	1.11
35°C	5.08	1.44–10.90	1.89
38°C	5.93	1.37–12.30	2.15
41°C	7.06	1.82–13.80	2.39
44°C	8.11	2.37–15.20	2.37

Table 1: Measured skin conductivities K at the arm and wrist, expressed in $\times 10^7$m.s^{-1}.

	Mean	Range	S.D.
N.H.	9.11	3.34–32.51	4.77
35°C	18.77	7.39–55.88	8.89
38°C	37.87	16.55–80.59	13.38
41°C	69.01	42.93–96.27	12.02

Table 2: nSkBF_{90} as measured at the arm, expressed in percentages (arbitrary unit).

References

[1] Euralliage. *2017 A (AW-AlCu4MgSi (A))*. 2023. URL: https://web.archive.org/web/20230214100923/https://www.euralliage.com/2017A.htm (visited on 02/14/2023).

[2] J.R. (Eds.) Davis. *ASM Specialty Handbook - Copper and Copper Alloys*. ASM International, 2001. ISBN: 9781615834532.

[3] C. H. Daly et al. "The Effect Of Pressure Loading on the Blood Flow Rate in Human Skin". In: *Bed Sore Biomechanics: Proceedings of a seminar on Tissue Viability and Clinical Applications organised in association with the Department of Biomedical Engineering, the Institute of Orthopaedics (University of London), Royal National Orthopaedic Hospital, Stanmore, London, and held at the University of Strathclyde, Glasgow, in August, 1975.* Ed. by R. M. Kenedi and J. M. Cowden. London: Macmillan Education UK, 1976, pp. 69–77. ISBN: 978-1-349-02492-6. DOI: 10.1007/978-1-349-02492-6_10. URL: https://doi.org/10.1007/978-1-349-02492-6_10.

[4] Alvin H. Sacks et al. "Difficulties in Laser Doppler Measurement of Skin Blood Flow Under Applied External Pressure." In: *Journal of rehabilitation research and development* 25-3 (1988), pp. 19–24.

[5] A. A. Anosov et al. "Volumetric Quantification of Skin Microcirculation Disturbance Induced by Local Compression". In: *Laser Physics Letters* 17.8 (June 2020), p. 085601. ISSN: 1612-202X. DOI: 10.1088/1612-202X/ab9445. URL: https://doi.org/10.1088/1612-202X/ab9445.

[6] J. C. Lawrence and J. P. Bull. "Thermal Conditions which Cause Skin Burns". In: *Engineering in Medicine* 5.3 (1976), pp. 61–63. DOI: 10.1243/EMED{_}JOUR{_}1976{_}005{_}023{_}02. eprint: https://doi.org/10.1243/EMED{_}JOUR{_}1976{_}005{_}023{_}02. URL: https://doi.org/10.1243/EMED%7B%5C_%7DJOUR%7B%5C_%7D1976%7B%5C_%7D005%7B%5C_%7D023%7B%5C_%7D02.

[7] A. R. Moritz and F. C. Henriques. "Studies of Thermal Injury: II. The Relative Importance of Time and Surface Temperature in the Causation of Cutaneous Burns". In: *The American journal of pathology* 23.5 (Sept. 1947), pp. 695–720. ISSN: 0002-9440. URL: https://pubmed.ncbi.nlm.nih.gov/19970955.

[8] Peter D. Wimberley et al. "Transcutaneous Carbon Dioxide and Oxygen Tension Measured at Different Temperatures in Healthy Adults." In: *Clinical Chemistry* 31.10 (1985), pp. 1611–1615. ISSN: 0009-9147. eprint: http://clinchem.aaccjnls.org/content/31/10/1611.full.pdf. URL: http://clinchem.aaccjnls.org/content/31/10/1611.

[9] Karim Bendjelid et al. "Transcutaneous pCO2 Monitoring in Critically Ill Adults: Clinical Evaluation of a New Sensor". In: *Critical care medicine* 33 (Oct. 2005), pp. 2203–2206. DOI: 10.1097/01.CCM.0000181734.26070.26.

[10] Aaron Conway et al. "Accuracy and Precision of Transcutaneous Carbon Dioxide Monitoring: A Systematic Review and Meta-Analysis". In: *Thorax* 74 (Sept. 2018), pp. 157–163. DOI: 10.1136/thoraxjnl-2017-211466.

[11] Food and Drug Administration. *Cutaneous Carbon Dioxide (PcCO2) and Oxygen (PcO2) Monitors - Class II Special Controls Guidance Document for Industry and FDA*. Tech. rep. U.S. Department Of Healsth and Human Services, 2002.

[12] G. N. Flint. "A Metallurgical Approach to Metal Contact Dermatitis". In: *Contact Dermatitis* 39.5 (Nov. 1998), pp. 213–221. ISSN: 0105-1873. DOI: 10.1111/j.1600-0536.1998.tb05912.x. URL: https://doi.org/10.1111/j.1600-0536.1998.tb05912.x.

[13] Monica Hindsén. "Metal Allergy: Aluminium". In: *Metal Allergy: From Dermatitis to Implant and Device Failure*. Ed. by Jennifer K Chen and Jacob P. Thyssen. Springer International Publishing, 2018, pp. 333–336. ISBN: 978-3-319-58503-1. DOI: 10.1007/978-3-319-58503-1_25. URL: https://doi.org/10.1007/978-3-319-58503-1_25.

[14] A. Fletcher Hall. "Occupational Contact Dermatitis among Aircraft Workers". In: *Journal of the American Medical Association* 125.3 (May 1944), pp. 179–185. ISSN: 0002-9955. DOI: 10.1001/jama.1944.02850210001001. URL: https://doi.org/10.1001/jama.1944.02850210001001.

[15] T. Peters et al. "Occupational Contact Sensitivity to Aluminium in a Machine Construction Plant Worker". In: *Contact Dermatitis* 39.6 (Dec. 1998), pp. 322–322. ISSN: 0105-1873. DOI: 10.1111/j.1600-0536.1998.tb05958.x. URL: https://doi.org/10.1111/j.1600-0536.1998.tb05958.x.

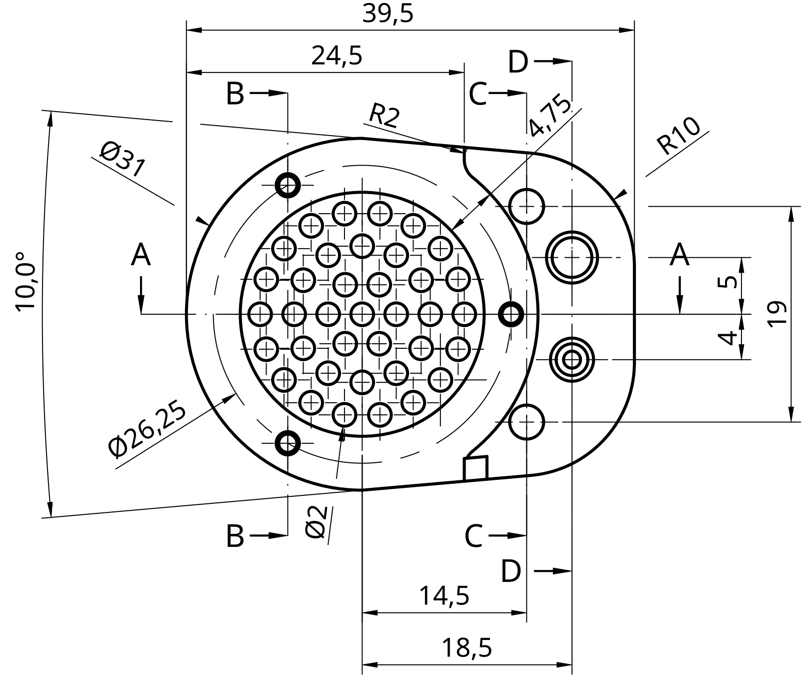

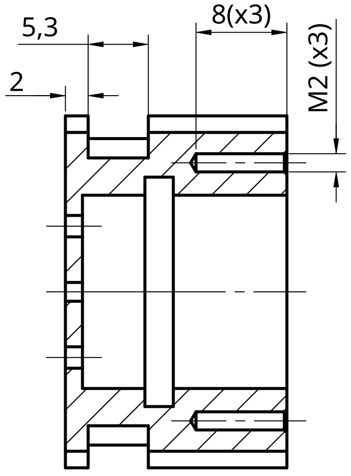

B - B

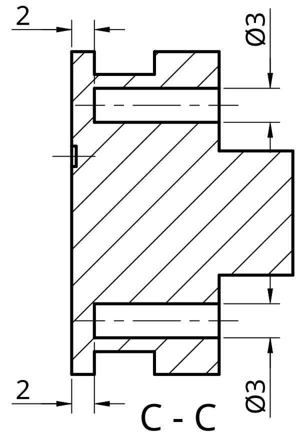

C - C

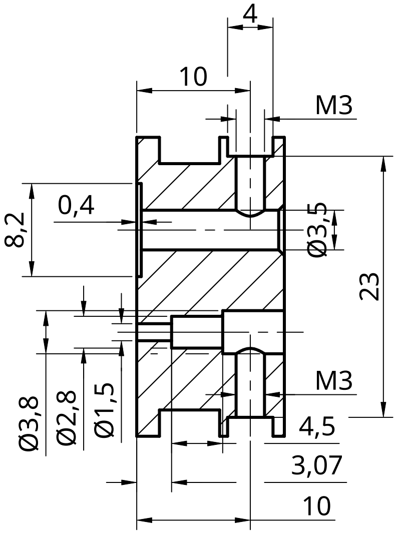

D - D

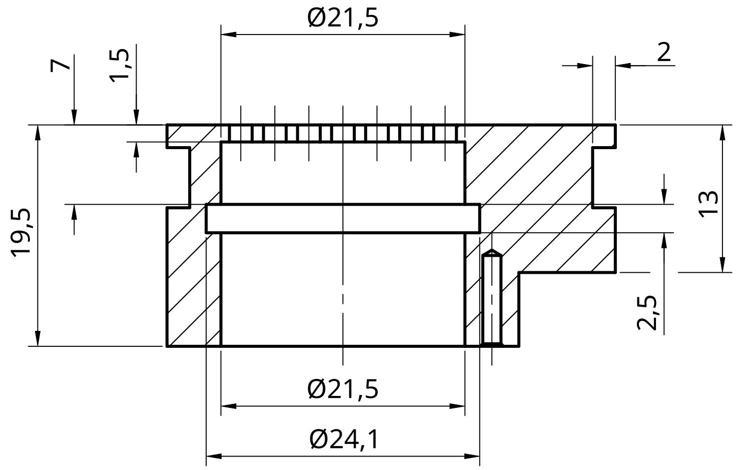

A - A

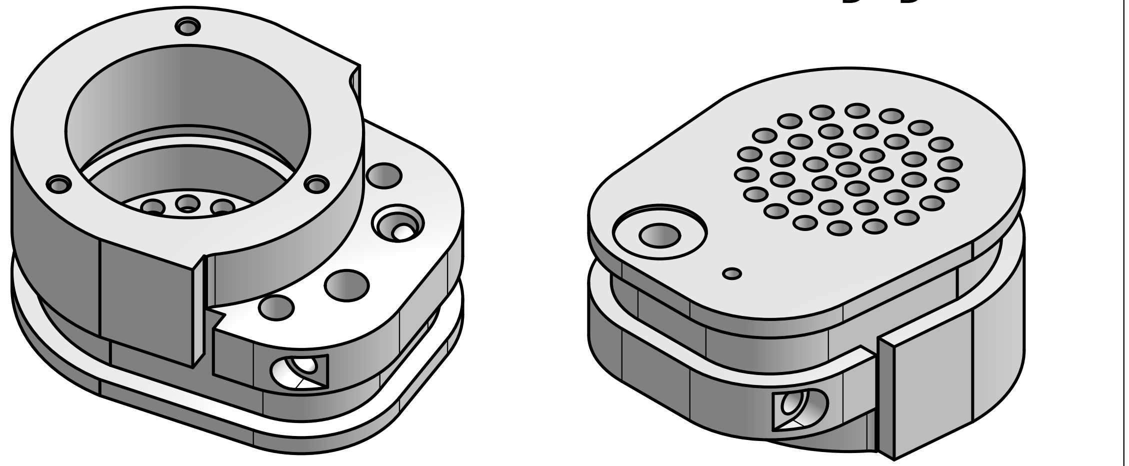

UNLESS OTHERWISE SPECIFIED, DIMENSIONS ARE IN MILLIMETERS<br>General Tolerance: ±0.1<br>Angular Tolerance: ± 0.1°<br>SURFACE FINISH		NAME	DATE	
	DRAWN	EMMANUEL DERVIEUX	2023/07/05	TITLE
	CHECKED			SENSOR BODY
	APPROVED			
DO NOT SCALE DRAWING				
BREAK ALL SHARP EDGES AND REMOVE BURRS				
FIRST ANGLE PROJECTION	MATERIAL<br>Aluminium 2017A	FINISH		SIZE A4 · DWG NO. 1 · REV. 1
				SCALE 3:2 · WEIGHT 26.27 g · SHEET 1 of 1
